# Midwifery

**Published:** 1825-04-01

**Authors:** 


### Midwifery.

1. *Puerperal Irritability.** Mr. Waller, the author of this paper, appears to have some obscure views of the principles we laid down in the second number of the present series of our Journal, pp. 415, *et seq.* He observes " every one who has been much engaged in midwifery practice, must have observed that there are two species of disease, equally dangerous in their nature, to which the puerperal female is obnoxious; in the one, the abdomen is affected, in the other, the head, (and this totally distinct from the puerperal mania.) This affection appears most frequently to take place after a copious loss of blood," though not invariably—and of the latter fact an instance is given. Mr. Waller concludes with the following observation. " My chief reason for publishing this case is to excite the attention of my professional brethren to a form of disease, which they must have every now and then met with, and which may be termed puerperal irritability, which is not noticed by authors, nor, as far as I am aware of, by our obstetric teachers, and which, consequently, is an exceedingly puzzling complaint to a young practitioner." Whether this affection be mentioned by authors, Mr. Waller and our readers will best judge by turning to the article in our second number, to which we have referred them. In point of fact, the cases here adduced are apt confirmations of the principles we have there laid down. We extract the last of these cases, because it is full of interest in itself, and because it is the more authentic from having been witnessed by Dr. Blundell.

" The subject of this case was a lady, ætatis thirty-seven, delivered of her first child after a short and natural labour, on the 25th of last month (October.) The placenta was retained, and, a very considerable hæmorrhage coming on, I judged it right to remove it, and in so doing found it to be adherent throughout the greater part of its extent. A very alarming state of syncope supervened previous to its removal, and continued for several hours afterwards, although there was but little after-hæmorrhage. She complained of intense pain at the præcordia,

* On Puerperal Irritability. By C. Waller, Esq. Med. and Phys. Journal, for February, 1825, p. 116.

which was relieved by administering a dose of laudanum in some warm gruel, to which a little essence of ginger had been added. She at length rallied, and appeared to be going on favourably till the 29th; with the exception, however, of weight and pain in the head, to which she had been subject for some time previous to her confinement. Her pulse was uniformly accelerated, seldom less than 100 in the minute.

"October 29th.—In consequence of being a little irritated, she has severe pain in the head, with throbbing of the arteries; her pulse is 100, and beats with considerable force.—Mittatur sanguis e brachio ad uncias viij. vel x. The blood presented an exceedingly buffy and cupped appearance; the coagulum was so firm that I could with difficulty pass my finger through it. This apparently trifling quantity of blood relieved the head greatly, but left her in such a state of syncope, that for many hours she could scarcely move hand or foot. Hyoscyamus was given to the extent of fifteen grains of the extract every four hours, with an occasional laxative. She slept much, but never waked refreshed, and there was uniformly a sensation of heaviness over the eyebrows.

"On the 2d of November (the ninth day after parturition, reckoning that of delivery as the first,) in consequence of a trifling domestic occurrence, she was thrown into a state of excessive irritability, and the symptoms of cerebral disorder, were much increased: she complained greatly of a feeling of constriction within the head, as if the cranium was too small for the brain, and it was on that account compressed. The senses of sight, hearing, smell, and taste, were painfully acute: the pulse about 100. Eight leeches were applied to the temples, and a laxative administered.—In the evening, finding her no better, I requested a consultation, and Dr. Blundell was sent for, when it was agreed to apply six more leeches to the temple, to give her the digitalis in operative quantities; to have the hair removed, and keep the head constantly wet and cold with a lotion composed of equal parts of spiritus eth. sulph. and water.

"November 3d (tenth day.)—Somewhat, though not very materially, relieved; pulse still at 100.—Repetatur remedia.

"Nov. 4th (eleventh day.)—The feeling of oppression still continuing, eight leeches were again applied to the temple.—Continuatur cetera.

"Nov. 5th (twelfth day.)—Was called to her about two o'clock this morning, and found the symptoms very materially aggravated: she had a weak, small pulse of 124; a whitish, but somewhat moist, tongue; a very pallid countenance; subsultus of the muscles of the lower extremity, and a quivering also of those of the lower lip; with that peculiar, faint, earthy smell of the breath I have more than once noticed in moribund patients. It was now very evident that, although action was increasing, that power was rapidly on the decline. I again requested the assistance of Dr. Blundell, and in consultation it was agreed to put her fully under the influence of opium, and to continue the evaporating lotion freely. This medicine was ordered in doses of three grains of the extract every hour, till sleep was procured. She began about six o'clock in the morning, and at one in the afternoon I gave her the sixth dose, (making eighteen grains of ext. opii in seven hours.) The pulse was now (one o'clock,) down to ninety-two, full, and round, very different to the irritable pulse of the morning, and she appeared in a very tranquil state. Another three-grain dose of the opium was given her at five o'clock, and a draught, composed of liq. am. acet. et mist. camph. every four hours. She slept all the night, and nearly the whole of the following day: her breathing was stertorous during the night, but she could be very readily roused. She wandered a little, but not much, during her sleep, and awoke very much refreshed.

"Nov. 6th.—Seemed considerably relieved this morning.—Repetatur haustus liq. am. acet; capiat pil. opii hora somni. This pill was twice repeated, as she seemed rather restless, which at length procured her some refreshing sleep.

"Nov. 7th.—Still on the mend, although she has had some slightly hysterical paroxysms, which distressed her greatly for the time. A little cascarilla infusion was now added to her draught.—Continuatur pil. opii nocte.

"Nov. 8th.—Convalescent: took two pills last night, which procured her sleep. From this time she has had no relapse, and is gradually, though slowly, regaining her strength. The secretion of milk was never entirely checked, nor was the lochial discharge.

"The foregoing case shows, in a striking manner, the utility of putting the system fully under the influence of opium, in this state of excessive irritability. Antiphlogistic medicines were had recourse to, with little or no benefit; but, so soon as this medicine began fairly to act, we find the pulse running down from 124 to 92: indeed, I think it very evident that we must have lost our patient but for this decided plan of treatment, as every thing seemed to indicate approaching dissolution." 121.

We shall leave this case after having pointed out several circumstances which appear to be particularly worthy of observation.

1. There are urgent symptoms of affection of the head after profuse hæmorrhage.

2. These symptoms were relieved by abstracting eight or ten ounces of blood, by which long continued syncope was induced.

3. They returned, as they had begun, after mental irritation.

4. They were finally subdued by opium.

Now we would ask, if hæmorrhage was the *cause* of this affection, was blood-letting the proper remedy?—is it not plain that the *head* was *relieved* only by the substitution and during the continuance of the *syncope*? does not the *effect* of the *opium* on the pulse and symptoms teach us not only what was the nature of the case, but what is the proper remedy in such cases? and we would wish to be informed what *good* could be anticipated from the digitalis.

---

2. *Ascites and Pregnancy.* Since we last noticed this subject in our third number, the following cases have presented themselves to our perusal in a foreign journal, from the practice of Dr. Olivier, the able author of a work on spinal diseases.

*Case* 1. Anne Chartier, 31 years of age, of robust constitution, was married in the year 1816. In April of the succeeding year, she found herself pregnant, and at the same time an ascites appeared, and became daily more distressing, till, in the 6th month of utero-gestation, the abdomen was of enormous size, the lower extremities being also greatly œdematous. She was forced to keep her bed during the three succeeding months, and was then safely delivered of a living, though a weakly child. One month after delivery she was tapped, and thirteen pints of clear water were drawn off. Both ascites and anasarca were cured by this operation, and the woman resumed her usual avocations. In the following December she again became pregnant, and again the ascites marched pari passu with the pregnancy. The lower extremities became enormously infiltrated, and the abdomen so distended as to threaten suffocation. She entered the Hotel Dieu of Angers on the 13th of July, 1818. She could not lie down in bed, and to her other distressing symptoms were joined severe head-ache, and frequent tendencies to lypothimia. During the next seven days the symptoms continued the same, in spite of remedies directed against the dropsy. On the 21st of July the orthopnœa was so pressing that paracentesis was imperiously demanded. It was performed by merely plunging in a lancet near the umbilicus. The water spouted out to the distance of five feet. It was limpid as distilled water. Sixteen pints were drawn off, and then the uterus could be distinguished. The patient felt greatly relieved. The fluid continued to ooze out of the wound for twelve days, and then stopped. The ascites again made progress, and on the 20th of August it was necessary to perform the operation as before, when eight pints of water were evacuated, and followed by the same relief. On the 22nd, labour pains were felt, and in three hours she was delivered of a living child. In fifteen days from this time the patient was discharged from the hospital, perfectly cured.

*Case* 2. A woman, 36 years of age, consulted Dr. Olivier respecting a dropsy of four years standing, which came on after an accouchement. A few months after consultation, the ascites had made such progress that paracentesis was performed—the patient relieved, and she went to her usual avocations. After this she became pregnant, and also ascitic, the dropsy forming an abdominal tumour of great prominence in the umbilical region, similar to the head of an adult. In this place, the patient performed paracentesis herself several times in the course of the pregnancy, by means of a writing pen, and she went on to her full time, the punctures healing kindly.—*Archives Generales, Oct.* 1824.

3. *Preternatural Presentation.** Mr. Brown has published a case of preternatural presentation, on which Mr. Thompson has made some severe strictures. We shall first state the particulars of Mr. Brown's case.

Mrs. Jackson, ætat. 30, of small stature, was taken with labour pains of her second child, at nine in the evening, (30th September) and at seven next morning Mr. Brown was in attendance. The pains being trifling he left her, but returned at four P. M. and, on examination, found the os uteri, fully dilated, the pains urgent—but these soon subsided. At five o'clock, the membranes being distended, Mr. B. ruptured them, and "*felt what he considered to be the right foot of the fœtus, with several inches of the funis umbilicalis accompanying it, on the pubic side of the superior outlet of the pelvis.*" No uterine action followed. At ten minutes before seven, he gave his patient an infusion of the ergot of rye. She began to complain of slight uneasiness in the belly and back —and in a short time the pain was not only urgent but incessant. Mr. Brown informs us that, after rupturing the membranes and making the examination, he endeavoured to bring down the foot, but could only get it just without the os externum. On this foot he used a considerable extractive force, but without producing farther descent. The other foot was, with some difficulty, brought down, and still more extraction made, but in vain. The labour pains were now urgent, and Mr. Brown discovered that what he had taken for the breech of the child was, in reality, its head, which was brought low into the vagina. Being here jammed, and all pulsation in the cord having ceased, our author sent for his instruments and, in consultation with Dr. St. Clare, performed cephalotomy, after which a very moderate degree of force on the feet delivered the fœtus. The mother recovered perfectly.

Such are the facts of the case, and we think they did not call for the severe animadversion of Mr. Thompson, who says it is "surprising to find a case so singularly, not to say *unprofessionally* treated." Hard words these Mr. Thompson—and not, we think, in the true spirit of Christian charity, or professional amenity. Mr. Brown, he avers, appears to have erred in three points.

"1st, In not, after rupturing the membranes, ascertaining the exact nature of the presentation.

"2dly, In using the ergot without such knowledge; and,

"3dly, In not resorting to the right means, which the above knowledge would have pointed out, at the time when practicable.

"It has been long laid down as a rule, from which we ought not to swerve, to endeavour to ascertain, on rupturing the membranes, the true nature of the presentation. This it is necessary to do, that we may be prepared, before the water has drained off, to employ any plan which its presence is likely to facilitate. To come at this knowledge, it may,

---

* Mr. Brown. Medical Repository, January, 1825. Mr. Thompson. Same Journal for February, 1825.

perhaps, be requisite to introduce the hand within the outlet, rather than leave the mind unsatisfied of the actual state of things. *It does not appear, in the report of the case, that this was done, but that the membranes were only ruptured, at five P. M., to hasten the contraction of the uterus: this was the ostensible reason, as we do not find that any correct idea was gained respecting the descending part, which might or might not be a foot with the cord.* That the knowledge was not correct, there is good reason to suppose; for we find that after considerable force, which had caused a further descent, the presentation above the part grasped by the hand was even yet judged to be the breech. When the true nature of the case was discovered, it was too late to remedy the error. Thus, then, by not being certain of the state of things early in the labour, what might have been comparatively simple was made complex, and, of course, the danger proportionally increased." 110.

Upon the above passage, we take the liberty of making a few remarks. From the latter part of the passage in italics, Mr. Thompson seems to aver that Mr. Brown made no examination after rupturing the membranes; whereas the latter distinctly states that he did examine, and found a foot presenting. Now as, *in most cases*, where a foot or feet present, we may expect that legs, thighs, and a breech will follow—and as Mr. Brown *did feel* what he thought was the breech of the child, he (Mr. B.) cannot fairly be accused of more than this mistake, which he candidly acknowledges, and which Mr. Thompson or any other practitioner might have made under similar circumstances. How very harsh then, and hypercritical is it, to condemn the candid narrative of such a mistake under the personal aspersion of *unprofessional treatment.* It is no wonder that unsuccessful cases and errors of judgment are carefully excluded from the public sight, when such comments as those of Mr. Thompson are to be the reward of candour! It is in this way that hypercriticism wounds the very vitals of medical science, by preventing the exposure of those rocks on which its votaries are daily wrecked! Delinquency in morals or medical ethics can alone merit the severe term of "unprofessional:"—to *errors in judgment,* it is unpardonable to apply such expressions.

That Mr. Brown may have been wrong in administering the ergot of rye, at the time he did, and without being perfectly certain of the position of the fœtus, we will not argue. We shall give the conclusion of Mr. Thompson's critique, as it involves practical matters of importance.

"It is apparent that the time was lost, when the case might have been made a simple one. There were two modes by which this might have been done. One, by risking the life of the child; the other, by which the child's life might have probably been saved. If at four o'clock the membranes had been ruptured, and the foot pressed up by the side of the head against the face, the case might there have become one of presentation of the cord merely. This method I adopted with success, in July 1817, in the case of a tailor's wife, in which the foot presented with the head, but without the cord: it was reduced with comparative ease. Dr. Ramsbotham, (as Mr. Brown might have informed himself, if he had desired, at page 300 of that gentleman's excellent publication,) describes a similar case, in which success followed the method pursued. But if the child had been turned either at four or at five P. M., its life would not have perished in all probability, and the woman, by doing so, placed in little or no jeopardy. At four the pains were active, and the delivery might have been effected immediately. At five, the feet might have been brought down into the vagina, and left there if thought necessary. After this time it was impossible to effect it with safety, as the waters were all drained off by walking across the room and the pains together. With the rest of the case I have nothing to do; it was effected with dexterity and despatch. I hope Mr. Brown will take what has been said in good part, because he has drawn animadversion upon himself; *for had he considered a moment, he would not have let either the observations or the case meet the public eye.*" 111.

We have already expressed our opinion on the principle conveyed in the last three lines of the above quotation. It is acknowledged, on all sides, that, in medical practice, the wisest and the most experienced are daily committing errors—yet here is laid down the Machiavelian precept —*never to let our errors meet the public eye!*

---

4. *Puerperal Fever.** From some interesting documents in the third number of our respected northern cotemporary, it appears that puerperal fever has been committing dreadful ravages in the Lying-in-Institution of Vienna, during the autumn of 1819. There were 56 dissections—an important basis for drawing pathological conclusions. The patients were mostly between twenty and thirty years of age. Most of them were of full habit of body, and only three emaciated.

"In all were observed many livid blotches over the whole body, and rapid putrefaction soon after death; in most of them immediately, so that in a few hours the bodies could scarcely be opened.

"In all there was a bloody discharge from the mouth and nostrils.

"In all the belly was more or less greatly distended, except in two, who had been delivered a considerable time before death.

"The parts of generation were every where more or less wollen, discoloured, reddish, and foul; four were syphilitic.

"Marks of blood-letting appeared in 13; of leeches on the abdomen in 7; of blisters alone in 17; and of both cupping and blisters in 12.

"In the head, the organs were always turgid with blood; the ventricles generally contained more than the usual quantity of serum; in other respects there was nothing worthy of notice in reference to this disease.

"In the trachea there was generally found a sanguineous fluid, and its internal surface was reddened.

---

* Baron Von Matoschex. Ed. Journal, No. 3, New Series.

"The lungs were always in the greatest state of expansion, turgid with blood, frequently adhering or united by effused lymph to the pleura, which was generally, but not in all, slightly red.

"In the cavity of the thorax and pericardium, there was invariably more than the usual quantity of bloody serum; the pericardium in no case morbidly changed, nor the heart externally, but its substance, without exception, more flaccid and tender than in the healthy state. Its internal surface, particularly the valves, chiefly of the right side, of a deep red, often of a black colour; the mass of blood generally fluid.

"In the abdomen there were only two cases in which there was no unnatural fluid, *i. e.* in those cases which had been delivered a considerable time before death. In all the rest there was found from one to two quarts of turbid, very fetid fluid, mixed with portions of coagulated lymph, and sometimes purulent matter; the latter appearance was observed in those cases where powerful antiphlogistic means had been employed, and which had survived longer after delivery.

"The peritoneum, omentum, and mesentery, exhibited, in five cases only, no appearance of redness; in the rest it was always somewhat more or less red, particularly towards the pelvis; and they were often agglutinated with the adjacent parts.

"With the exception of two cases, the stomach and intestines were always much distended with air, and their external surface more or less red. *Lumbrici* frequently appeared in great numbers, not only in the small, but also in the large intestines, in the stomach, rectum; and, in one case, in the nostrils.

"The liver and spleen were always similarly affected; they were much more pale, flabby, and tender than in their healthy state; easily broken down with the finger, similar to the degeneration of the uterus, and filled with somewhat fluid blood; These appearances were seen even in those two cases where the abdomen was not distended, nor contained fluid, and in which the intestines were healthy. The gall-bladder was always much filled with dark bile. The pancreas always healthy.

"The kidneys, in most of the cases, were flabby and tender. The ureters always somewhat enlarged, and red. The urinary bladder always contracted.

"The internal parts of generation were every where covered with yellow coagulated lymph, except in the two cases, in which no fluid was found in the abdomen; and in those cases in which strong antiphlogistic means were used, there was frequently a thick yellow purulent fluid often externally on the neck of the womb. The ovaria and Fallopian tubes were always more or less swollen, red and tender.

"The uterus, in all cases little contracted, was more or less red externally, even in those where the delivery had taken place long before, and the abdomen was not otherwise in an unhealthy condition. The substance or body of the uterus was always flabby, tender, easily broken down by the finger; in two cases, full of small holes or cavities filled with stinking blood. In two cases, the uterus, on account of the tenderness of its substance, had burst, during delivery, at its neck; in the one case, the rupture was four inches in length; and, in the other, one inch and a half. The cavity of the uterus was found filled with fetid air several times, particularly in the syphilitic women. Its internal surface appeared generally covered with offensive cineritious ichor or mucus, only seldom with offensive viscid blood. Beneath this, it was always red, discoloured, often as if slightly eroded or ulcerated, the internal membrane very much eroded, and destroyed. Thus, in a woman of eighteen years of age, who had been delivered three weeks, in whom the abdomen was not distended, nor contained fluid, and there was nothing diseased in the abdominal cavity, except the liver and spleen being flabby and tender, the uterus was entirely ulcerated on its internal surface, like the internal surface of one of the large intestines, when covered with ulcerations. She died on the twenty-ninth day. On the fundus of the uterus, there generally remained particles of the placenta and *membrana decidua*, in the form of putrid shreds. The colour of the cervix of the uterus was constantly darker than the rest; and it always was darkest, and penetrated into its substance, at the mouth of the womb, which was likewise much dilated, and often as if destroyed with loss of substance, often cracked, as if rent. The vagina was generally preternaturally red.

"Such were the facts carefully abstracted from the whole fifty-six dissections." 86.

From these and other phenomena about to be mentioned, we think it is evident that this puerperal epidemic of Vienna, differed considerably in its pathological characters, from the puerperal fever which we are accustomed to see in this country. Whether these modifications depended on the nature of the disease, the constitutions of the patients, the climate of the place, or the modes of treatment, we cannot pretend to determine. A committee being appointed to investigate the nature of this dreadful scourge, the work was set about with more zeal than success. According to Professor Boër, the accession of fever was always preceded by some deviation from health—particularly in the uterine system. The lochia disappeared either immediately, or in a few hours—the mammæ became empty, loose, and flabby.

"But the most alarming circumstance was, that while the disease was yet, in all appearances, a recent acute inflammation, Dr. Boër, on examination with the finger, already discovered, in the mouth of the uterus, marks of gangrenous disorganization, which were rendered evident even to the by-standers, by the putrid smell of the finger." 87.

Such a phenomenon as the above we do not recollect to have noted in any English author on puerperal fever.

The last stage of the disease presented all the symptoms of effusion in the chest and abdomen—which symptoms were amply confirmed by dissection. All the viscera were found in a soft and putrid condition, while the blood was in a dissolved state. The internal surface of the uterus was destroyed by gangrene—particularly about the os uteri. This was the case in the whole of the 56 dissections. The treatment was completely unsuccessful, as the following extract will shew:—

"The treatment of the disease, according to the report of Professor Boër and his assistant, confirmed by Dr. Raimann, had hitherto been chiefly antiphlogistic. They employed in most cases, immediately on the commencement of the disease, repeated venesections, the application of leeches, emollient cataplasms, emollient clysters; at a later period, blisters, with the corresponding internal remedies; in some cases calomel and other celebrated remedies; and in some, where gastric affections at first predominated, emetics and other appropriate remedies were given. On the commencement of debility, they had recourse to diffusible stimulants, but always with the same unfortunate result; the only difference being, that some were carried off by the violence of the disease very quickly, even in a few days, and one in six hours, others more slowly. The chief physicians, Schiffner, Festi, Belletzky, and Director Raimann, were not less unfortunate in the treatment of those pregnant women whom they took out of the Institution into their respective divisions of the Hospital. Notwithstanding the meritorious and scientific exertions of these able practitioners, all of their patients died." 88.

The causes of the disease could not be ascertained by the committee. No contagious property could be traced by Professor Boër. The committee, however, entertained doubts on this subject.

Upon the whole, these documents, triste and authentic as they are, throw little or no light on the nature or treatment of puerperal fever—nor do they hold out any increase of hope in medicine, when one of those dreadful epidemic scourges has once commenced its destructive career.

---

5. *Supposed Scirrhus Uteri.* In the Revue Medicale for March, there is a case which is said to be scirrhus of the uterus successfully treated by iodine, internally administered. We mention the case, because we have seen many instances where pain and morbid sensibility had taken such possession of the cervix and os uteri, as to lead to the belief that the parts were organically affected, but where, in fact, the case was much otherwise. These complaints are not described by obstetric authors, in any clear manner. They might be called cases of IRRITABLE UTERUS, or perhaps HYSTERALGIA; and are well worthy of a monograph from some experienced accoucheur. We understand, indeed, that Dr. Gooch will favour the profession with some observations on this subject. We had the advantage lately of his advice, in consultation, on two cases of this kind, one which is recovered, and the other is under treatment.

Dr. Klaproth's patient was only 23 or 24 years of age, and after two miscarriages and obstructed menstruation, became affected with leucorrhœa, pain in the groins, and sense of heat in the vagina. On examination, he discovered *an induration at the os uteri, accompanied with sensibility and heat.* This is all the evidence he gives of scirrhous uterus. Eight drops of the tincture of iodine were given thrice a day, and an ointment, containing digitalis, belladonna, and hyosciamus, externally applied. The patient soon menstruated, and, in process of time, all the symptoms of scirrhus, if such they could be called, vanished. We consider the above as a case of IRRITABLE UTERUS.

5. *Tendency to Plethora in the pregnant State**. In the second number of this series (Sept. 1824, p. 355, *et seq.*) we drew the attention of the profession to a paper entitled, "*Observations on a Species of Premature Labour, to which Pregnant Women are not unfrequently liable,*" by an experienced physician. The paper of Dr. Musgrave, now before us, bears on this important point, although it was drawn up before our journal reached Antigua. We beg our readers first to recur to our analysis of the paper alluded to, and then they will profit more by Dr. Musgrave's case, which we shall now endeavour to abbreviate.

*Case.* Mrs. R. M. now 27 years of age, of a robust form, was married in October, 1814, and twice miscarried at an early period afterwards. On the 7th of January, 1817, she was taken in labour, at the full period, and was delivered by forceps in the midst of convulsions. By bleeding and other proper means, she recovered, and the child lived.

In 1818, the lady again proved pregnant, and was safely delivered on the 21st of March, 1819, having been annoyed in the latter months of utero-gestation, by a sense of pain and fulness in the head, for which she was twice blooded, and the bowels carefully attended to. Shortly after the expulsion of the placenta, this time, she suffered acutely from hysteralgia, till relieved by an opiate. To this succeeded a discharge, nearly amounting to hæmorrhage, requiring cold applications, &c. Ten or twelve days after delivery, she insisted on having a tooth extracted; a few days after which (having been exposed to cold) she complained of pain extending from the face to that side *(right)* of the head. Liniments—anodynes—purgatives—antimonials, appeared to give relief for a time. But, on the second night from this time, she was found with symptoms of slight apoplexy, and with complete hemiplegia of the *right* side. Bleeding, blistering, and purgation dissipated the cerebral symptoms, but the paralysis was long in subsiding, and the right arm, to this day, is partially paralytic.

Again the lady became pregnant, and between the second and third month, while sitting at supper, she was attacked with severe pain in the *right* side of the head, sickness at stomach, and confusion of ideas. These were quickly relieved by evacuations, and her usual health was restored. It was at this time, that the foregoing history of her case was drawn up by Dr. Musgrave, and transmitted to several eminent practitioners in Great Britain, together with the following queries.

"1. Was or was not the hemiplegic attack connected with the puerperal state?

"2. Could the extraction of a tooth at that particular time have had any and what influence in producing the attack?

"3. Was the plan of treatment adopted by Dr. Musgrave defective in any and in what points?

"4. What regimen and what remedies will, at this period, be

* Dr. A. Musgrave, of Antigua. Med. Repos. Feb. 1825.

most likely to carry Mrs. R. safely through her pregnancy, as well as lead to restore the lost power over her hand and wrist?" 93.

We can only introduce the opinions of Drs. Hamilton of Edinburgh, and Davis of London, on the above case—the other opinions, indeed, agreeing, on all essential points, with those detailed by the gentlemen in question.

*No. 1. Dr. Hamilton's opinion.*

"In reply to the queries stated by Dr. Musgrave, I take the liberty to offer the following as my unbiassed sentiments:—

"I. The hemiplegic attack appears to me to have been connected with the puerperal state, both because that state predisposes to affections of that description, and because Mrs. M. had already had a decided modification of the same cause which produces hemiplegia.

"II. The mere extraction of the tooth could not have been the cause of the hemiplegia; for had it been injurious, it should have produced convulsions; but the subsequent inflammation of the gum, with its extension to the neighbouring parts, from its effect upon the nerves which proceed from the base of the skull, appear to me to have been the probable cause of the hemiplegia.

"III. The plan of treatment adopted by Dr. Musgrave seems to me to have been, in every respect, most active and judicious; and I have no hesitation in affirming, that but for the decided bleeding on the 7th January, 1817, and the prompt extraction of the infant, Mrs. M. could not have survived the delivery. As to the attack of hemiplegia, it could not have been foreseen, and I scarcely believe that it could have been prevented after the incoherence of ideas was discovered. From the account of the case, the state of the pulse did not indicate blood-letting; and I am almost certain that the subtraction of blood at that time could not have stopped the progress of the disease.

"IV. The means which appear to me most likely to conduct Mrs. M. safely through her present pregnancy, to prevent the recurrence of the alarming symptoms, and to restore the use of her hand and wrist, are the following:—

"First, Her diet should consist entirely of vegetables, with the smallest possible proportion of liquids, and with total abstinence from all fermented liquors of every description.

"Secondly, she should make as little exertion as possible, one great object being to keep the action of the heart and arteries perfectly quiescent. On this account, she should indulge very much in the horizontal posture; and, for the same reason, every circumstance which may tend to excite any sudden or violent emotion of the mind, is to be scrupulously guarded against.

"Thirdly, The bowels are to be opened twice or thrice daily. For this purpose one or two of the laxative pills (A) are to be taken every night at bed-time, and a suitable dose of the sulphate of magnesia, or the sulphate of potass, or the pulv. salin. comp. of the Edinburgh Pharmacopœia, every morning fasting.

"Fourthly, One of the powders (B) dissolved in half an English pint of boiling water, and allowed to cool, is to be taken daily, in divided prises, in the course of the forenoon. This medicine produces no sensible operation, but it has, for several years, seemed to me to have an especial influence in removing topical congestions or thickenings within the cranium."*

"Fifthly, Should any symptoms of interrupted circulation within the head threaten to occur, blood ought not only to be drawn from the arm, but also from a branch of the temporal artery.

"Sixthly, If labour be at all protracted, venesection must be had recourse to.

---

"* Will Dr. Hamilton forgive me for acknowledging that I never for an instant, after having read it, contemplated availing myself of his prescription (B) and that I have ventured more than once, since the receipt of his opinion, to combine the submuriate of mercury with other medicines, when it appeared to me that the state of my patient's bowels required the administration of an active purgtaive?

"To his admirable course of instruction again and again attended, I refer with gratitude, the groundwork of any knowledge of midwifery I may happen to possess; and I have, consequently, been accustomed to regard his doctrines on subjects allied to his peculiar department, with a species of respect amounting almost to veneration. But, at a distance from the schools, and to a certain extent, from books, I have learned, during a practice of ten laborious years, to think occasionally for myself, and to shake off that slavish deference for authority, which too often characterises the outset of a young man's professional career. I have therefore thought attentively on the Doctor's suggestions; but no exercise of deliberation has enabled me to comprehend either the circumstances which led him originally to conjecture, or the basis on which he could afterwards have founded the more settled conviction that a combination of borax and cream of tartar (two of the simplest articles of the materia medica), which produces, he admits "*no sensible operation*," and requires to be continued *for three or four months*, has "an *especial* influence in removing *topical congestions* or *thickenings within the cranium*."

"It may be urged that a prescription of this kind is, at all events, harmless. In *itself*, I freely grant that it is so, but not in its *consequences*, emanating, as it does, from such powerful authority. *These* may be not only mischievous, but *fatal*, by diverting the attention of the inexperienced from more efficient and less questionable means. But it is too obvious that even this brief opinion savours most strongly of those lamentable prejudices which so generally pervade that memorable volume wherein the virtues of the muriate of lime (which, by the bye, likewise "produces no sensible operation, and may be continued for many months, not only with perfect safety, but also without any inconvenience or restriction whatever,"*) are lauded to the skies; and the safety of prescribing calomel in croup—a practice for the more general introduction of which we are indebted to himself—is most seriously called in question.

"That grave men should violently persist (to use *partially* a quotation of his own from Professor Carlisle) in maintaining *prejudices so absurd*, is passing strange. Men starting into the exercise of the medical profession from a cloistered study of books, and from abstract speculations—men wholly unaware of the fallibility of medical testimony, and unversed in the doubtful

* Observations on the Use and Abuse of Mercurial Medicines, p. 115.

"Seventhly, After delivery no opiate should be given; but camphor, to the extent of from one to two scruples per diem, in the form of julep, as per recipe annexed (C), ought to be exhibited so long as the lochial discharge continues.

"Eighthly, The diet during lying-in must be regulated very much by circumstances. Mild animal food may probably be useful; but wine or other stimulants had better be avoided.

"Ninthly, If, after delivery, the use of the right hand and wrist be not restored, it may be worth while to try another course, *for three or four months*, of the powder (B), and to apply a succession of blisters to the nape of the neck.

"Lastly, Every preparation of mercury ought to be refrained from; and if, unfortunately, hydragogues or similar remedies be required, preparations of antimony with gamboge, or the compound powder of jalap or hellebore, are to be employed.

"The foregoing detail renders it unnecessary for me to make any farther remarks on the treatment pursued; but I cannot forbear adding, that I have very seldom received so accurate a detail of a case, and that I have still more seldom had occasion to approve so unreservedly of the practice pursued." JAS. HAMILTON, JUN. M.D.

Edinburgh, 23, St. Andrew's Square,
September 24th, 1820.

FOR MRS. M.

(A) ℞ Extract. Hyoscyam. Nigr. ʒss.

——— Colocynth. Comp. ʒj.

Ft. massa divid. in pil. æqual. no. xxiv. Sign. Laxative pills.

(B) ℞ Sub-Bor. Sodæ,

Supertart. Potassæ, āā ʒj.

Ft. dos. et mitte tal. no. xxiv. Sign. Powders, one to be taken only as directed.

(C) ℞ Camphor. ℈ij.

Alcohol. gtt. xxx.

Sacch. Alb. ℥j.

Carbon. Magnes. ʒj.

Tere simul. probè, et adde paulatim Aq. fervent. ℥viij. Ft. julep. Sign. Two table spoonsful a dose.

J. H. JUN.

September 24th, 1820.

---

effects of medicines—may be themselves deluded, and delude others for a time; but when experience has proved their errors, it would be magnanimous, and yet no more than just, to renounce them."

"Upon this noble principle, I am not altogether without hope that I shall yet see an ingenuous recantation on the part of my respected preceptor, of those fanciful opinions, which he must ere this have discovered, are viewed by his stanchest adherents as spots that tarnish the lustre of his otherwise brilliant reputation."

No. 2. *Dr. Davis's Opinion.*

29, George Street, Hanover Square,
October 2, 1820.

"In the time allowed for the drawing up of an opinion upon the case of Mrs. R. M. and especially on this day, it is not possible for me to enter into so minute a discussion of its several bearings as its able author appears to expect. The following remarks will contain briefly, indeed, but I trust distinctly, the results of my reflections upon it:

"1. I think the attack of hemiplegia was so far connected with the puerperal state, that probably it would not have taken place, had that state not been present at the time.

"2. The extraction of the tooth and its consequences, viz. the effects of the imprudent exposure to the action of cold, might have co-operated with a previously existing strong predisposition to cephalic congestion to produce the disease.

"3. Women are, in some degree, liable to apoplexy and paralysis, during the puerperal state. Dr. Orme met with five instances of sudden deaths at this period, during his life of obstetric practice in London. Dr. Sims has seen three or four cases. I have met with two myself, together with four or five of apoplectic seizures and consequent hemiplegia. In almost all my cases, there existed that form of body which is considered as predisponent to diseases of this class: and I believe I may assert, that in every case, without an exception, there was more than an ordinary relish for food.

"4. I have met with several cases of partial paralysis from imprudent attempts to suckle without the ability. These cases have yielded usually to tonics and the tepid bath, nursing, of course, being abandoned.

"5. The treatment of the convulsions during Mrs. M's first labour was perfectly proper.

"6. The plan of treatment of the second labour, and of its consequences was, also, in my opinion, equally judicious, though less decidedly successful. I have given my sanction more than once to the extraction of a tooth during the convalescence after parturition. Dr. Sims (whom I met last night in consultation, and to whom I mentioned the interesting case of Mrs. M.) has often done the same. The extraction of the tooth should be considered separately from the exposure afterwards to the action of cold. The medical attendant was professionally answerable for the consequences only of the former. It is not to be presumed that the extraction of a tooth *per se* could materially conduce to occasion cephalic congestion: however, it might indirectly lead to it afterwards, in the event of mismanagement or neglect.

"7. With respect to the indication for earlier bleeding as a preventive measure, suggested in page 6th of the case, it is impossible for me to give a decided opinion. I can only say, generally, that we are not in the habit of treating the irritations and reactions now and then occurring after the extraction of a tooth by so active a measure as venesection; and I would observe further, that in the circumstances related in this case, it is very probable, and *almost certain*, that I should not have thought of such a thing in my own practice.

"8. The entire sequel of the treatment has been so active and varied and judicious, that it has scarcely left room for an additional suggestion—much less an improvement on it.

"9. As to the regimen and remedies to be recommended, my opinion, will strictly coincide, I am persuaded, with that of Dr. Musgrave. The general functions of the system should be kept as well balanced as possible. Any local *visceral* derangement, however temporary, will endanger the head. All causes of visceral derangements, should, therefore, as much as possible, be avoided. There is obviously a remarkably strong disposition to cephalic fulness. The state of the circulation should be frequently and nicely examined. Pregnancy is itself a plethoric state, in most cases, as well as often a state of irritation. Venesection is the most certain remedy of both states. The bowels should, of course, be kept in a soluble state. Small doses, daily or frequently, of neutral salts or magnesia, would perhaps meet this indication conveniently. The patient, if in moderate health, should take moderate and regular exercise—scarcely up to the point of fatigue, and never beyond it. As for food, it should be very sparing, and should consist principally, if not entirely, of vegetable matter. This important measure of precaution must be attended to at the expense of any amount of inconvenience to the patient. As to suppers, they should be prohibited altogether; and on no account should meat be indulged in more than once a day; but I think it would be the safest plan to forbid animal food altogether.

"10. I am at a loss what to say as to the probable issue of the paralysis still remaining. I am, however, upon the whole, inclined to the opinion that it will eventually yield to the influence of time, assisted by the tepid bath and local stimulants; but electricity, in every form, should be avoided at present, as being calculated to induce premature labour.

"Should the above remarks serve the interests of the patient in any degree that may tend to the improvement, and finally perfect establishment of her health, I shall feel much gratified. They would have been more extended, had more time been allowed."

DAVID D. DAVIS, M.D.

These queries were sent off in the lady's third month of utero-gestation, and we must take up the case again, from that epoch. Dr. Musgrave, after evacuations had been premised, confined the patient to most abstemious regimen. She went on well—quickened at the usual period, and shewed nothing unsatisfactory till the end of the sixth month, when, after some mental agitation, the motions of the fœtus were felt very violent, after which they became faint, and ceased altogether. But as the patient had no uneasiness in the head, back, or elsewhere, Dr. M. did not think himself justified in giving any other advice than caution to keep herself quiet. One week after this period, the patient complained violently of pain in the right side of the head, with sense of intolerable oppression there. Her pulse, to Dr. M's astonishment, was only 37 in the minute, extremely full—" and with repeated and awful intermissions." Blood was instantly drawn from the arm, *pleno rivo*, and some relief was experienced by the time that forty ounces were abstracted. The action of the heart then became less oppressed and irregular. She requested, however, that the venesection might proceed, and blood was accordingly drawn till the pulse rose to 84, " with a corresponding improvement in every other respect." The quantity drawn amounted to 62 ounces. Purgatives were administered, and cold applied to the head. At nine o'clock the same night the pain again returned, and with it a diminution in number of the pulse, with some intermissions. In consultation, it was agreed to bleed again, and eighteen ounces were abstracted before satisfactory results were procured. Parturient symptoms manifested themselves in the evening—the shoulder presented—turning was effected, and a dead fœtus, of 6½ months, was extracted. The patient appeared to derive the greatest relief from the lochial discharge, and next morning declared herself free from complaint. In the evening. Dr. M. was summoned, and the patient declared her conviction that she was dying, " from that same sense of intolerable but defined oppression which had ushered in this attack, and her pulse was, as before, awfully slow, full, and intermitting." A vein was instantly opened, and thirty ounces of blood were abstracted before complete relief was obtained. There was no recurrence of untoward symptoms, and Mrs. M's convalescence was unusually rapid.

Now that the bustle was past, Dr. M. began to ruminate on the complaint and treatment, and he entertained some doubts, whether he ought not to have bled his patient when she first complained of the cessation of motion in the fœtus.

Mrs. M. was very shortly re-established in perfect health, with no vestige of her past sufferings left, except the weakness in her right hand, which still remained. She persevered in the most abstemious regimen, avoiding every kind of stimulus—keeping her bowels regular with the aloetic pill—and taking only such exercise as might conduce to health, without suddenly increasing the action of the heart. Under these severe restrictions, it was astonishing how speedily she regained flesh, strength, and spirits.

Nothing particular occurred till the beginning of 1821, when Mrs. M. again became pregnant, and in a few weeks afterwards, gave indications of cerebral fulness, which was removed by the lancet. All went on well till the close of the 7th month, when she announced a cessation of the active motion of the child, accompanied this time by a slight sense of weight within the head. Venesection was immediately had recourse to, and relieved the head: but labour came on in another week, and a putrid fœtus was expelled.

The lady continued tolerably well, with the assistance of one venesection, till December, 1822, when she became pregnant, but aborted at the end of two months. Her recovery, as usual, was rapid and complete. In a few weeks, the 8th conception was announced, and the eighth month of it completed, without the aid of the lancet. Within about a fortnight of the full period, according to her own reckoning, Mrs. M. complained of the same lateral and circumscribed head-ache which had so often menaced her with death. Our author was sent for, and found the pulse as low as 44, full and intermitting. She was bled immediately, and relieved effectually. Eight days after this period, labour came on, and a small dead child was expelled. After this confinement, Mrs. M. suffered a severe attack of phlegmatia dolens, which gave way, at length, to the usual treatment.

This long eventful history is now near a close. In March of 1824, this lady required a large bleeding, on account of threatened mischief about the head. In May, she again conceived, for the *ninth* time, and miscarried in June. In August of last year, she was troubled with occasional sense of weight over the right eye, and at length observed that, on awaking in the night, she could neither articulate nor remember her husband's name. The loss of about eighteen ounces of blood produced faintness, and removed all complaint.

"Professor Burns, of Glasgow, says, in speaking of impracticable labour: 'How far it is proper for women in these circumstances to have children, is not a point for our consideration, nor in which we shall be consulted. I would say that it is not proper: but it is no less evident that when they are pregnant we must relieve them.' The remark applies with equal force to the case under review: and it would, indeed, be infinitely gratifying to me, could I elicit the observations of the able and experienced, through the medium either of the Medico-chirurgical Review, the Medico-chirurgical Transactions, the London Medical and Physical Journal, the London Medical Repository, or the Edinburgh Medical and Surgical Journal—all which periodical works are regularly forwarded to me through the post by the first monthly mail after their publication. I would propose for attentive consideration the following queries:—

"1. To what is the premature death of the fœtus in utero, in this case, to be attributed?

"2. By what means is it probable that the state of the system may be so modified as to prevent this occurrence in future?

"3. Has Dr. Musgrave been guilty of any error, either of *omission* or *commission*, in the treatment of this case for the last four years; and *especially, ought premature labour to be induced by art in the event of any future pregnancy exceeding the term of seven calendar months?*

"It may be as well to mention that Mrs. M's menstrual discharge, when in ordinary health, is tolerably regular in its returns, and natural in quantity and duration, although her periods seldom reach to the full extent of four weeks. A. MUSGRAVE, M.D."

"St. John's, Antigua, 4th November, 1824."

"P.S.—I had entirely closed my communication when the Medico-chirurgical Review for September (No. 2. of the New Series) came to hand. The extract inserted at page 356, from "the Observations of an Experienced Physician,"* it will be obvious, delineates with accuracy the circumstances of Mrs. M's situation.

"I have not time just now, as the mail will be made up to-morrow, to enter at length upon a discussion, from which I had purposely abstained, in detailing the facts of this case. Suffice it, therefore, to state, with reference to the queries submitted in these observations, and the replies of Dr. Beatty, that the suspicion of a venereal taint in the parents having acted upon the fœtus in utero, is here *entirely out of the question*. I have known the husband from infancy, and his moral character I have no hesitation in affirming to be indisputable. The two children now living enjoy, and have always enjoyed a share of health which cannot be exceeded under any circumstances, or in any climate; and had the father been infected since their birth, I am persuaded, from the terms of close intimacy which subsist between us, that the occurrence neither could nor would have been concealed from me. The exhibition of mercury, therefore, would be beyond measure absurd.

"That 'more blood has always circulated in Mrs. M's system than was consistent with the health of the fœus, is beyond all doubt: and it only remains to be ascertained in parallel examples in what particular manner this plethoric tendency proves destructive, and by what means more judicious than those devised by me, this state of the system may be successfully corrected.

"I have, of course, indulged in my own speculations; but as I do not feel myself competent to *convey*, my object in giving this history to the medical public was exclusively, as I stated, to *elicit* information, as I was not aware, till it was concluded, that the attention of the profession had been already pointedly attracted to this particular species of premature labour.

"It is abundantly evident that Mrs. M's case furnishes a remarkable proof, in addition to those enumerated at page 446 of the number of the Medico-chirurgical Review already adverted to, that paralysis and cerebral lesion may be occasionally on the same side." 109.

We hope Dr. Musgrave will elicit some observations from our obstetric brethren on this point. Dr. Beatty's opinion on the case in question would assuredly be gratifying to Dr. Musgrave, and we trust he will be favoured with it through some of the channels pointed out. We have only one remark of our own to make, and that is, that because Dr. M. has no reason to believe that any syphilitic taint may be acting a part in this drama, "the exhibition of mercury would be, therefore, beyond measure absurd." We are far from thinking that a very gentle and cautiously administered course of mercury would prove

---

* Transactions of the Association of Fellows and Licentiates of the King's and Queen's Colleges of Physicians in Ireland, vol. IV.

prejudicial to this lady's health, allowing there was no possibility of syphilitic taint. On the contrary, we should be inclined to such a measure, on account of the often-recurring tendency to cerebral affection. Nothing would have a greater chance of equalizing the circulation and producing a regular and healthy state of the secretions—and nothing, in our humble opinion, would give the lady a better chance of becoming the mother of a living child—*provided that event be desirable.* To this last question, we will not take upon ourselves to offer any answer; but we may be permitted to hint our belief that, all things considered, there does not appear to us to be so much to dread in a regular and full termed delivery, as Dr. Musgrave seems to apprehend, remembering that the lady has already borne two full-grown living children, and has a well-formed pelvis. But we leave the case to the experience of our obstetric brethren.

---

6. *Substitute for the Cæsarean* Section. M. Baudeloque, the younger, has published in the Journal Universel, for July last, a proposal of this kind. The first incision is to extend along the outer border of the rectus muscle, from the umbilicus to within two inches of the pubis, not through the peritoneum. The waters are next to be let off, by rupturing the membranes, per vaginam. The thighs and legs being then half bent, the operator passes the fore finger into the inferior angle of the wound, removing the peritoneum carefully from the whole iliac fossa—and, when detached, one assistant raises up the peritoneum and mass of intestines, whilst another maintains the uterus in its situation, by applying one hand on the belly. The operator is then to introduce his right hand into the abdomen, so as to explore the iliac artery, and ascertain if there be any arteries surrounding the vagina. "If there be any, I apply (says M. B.) a ligature at both extremities; and before cutting them, I take care to recognise the sub-pubian ligament, in order to avoid it." The bladder being emptied by the catheter, and the rectum by glysters, the left hand, smeared with lard, is to be introduced parallel to the axis of the inferior orifice, when the vagina will be easily brought out beyond the external wound. Seizing the bistoury, he covers it with the three first fingers of the right hand, and plunges it into the vagina by the external opening, and as far as possible below its insertion into the neck of the uterus, prolonging the incision to the extent of four inches and a half. This incision made, the fœtus is expelled, by the contraction of the uterus, into the abdominal cavity, in the same way as happens in rupture of the uterus or vagina. It may be extracted with forceps.

Another mode of operating differs from the above only in respect to the peritoneum, which is divided at the same time with the abdominal parietes. The rest of the operation is rendered more easy in consequence of the section of the vagina being thus capable of greater extension.

We shall only remark that the above operations appear to us most horrible, difficult, complicated, and, we venture to prognosticate, impracticable.

---

7. *Dropsy in Pregnancy.* Dr Felici, of Milan, relates the case of a woman, 35 years of age, who considered herself pregnant in December 1817, soon after which a tumefaction appeared in the feet, and spread to the thighs, pudenda, loins, and abdomen, attended with much thirst. On the 2nd February, she was seized with fever, and acute pain in the left hypogastric region, with numbness of the corresponding thigh. These symptoms were combated by antiphlogistics and digitalis, except the thirst, which continued insupportable. The œdema increased—the swelling of the abdomen became enormous—the desire of making water violent and painful. The pain in the leg and thigh had greatly increased—there was difficulty of breathing—palpitations—a dry cough—wasting of the upper extremities—frequent pulse—sense of suffocation upon the slightest motion—puffiness of the face. Such was the poor woman's state when seen, for the first time, by Dr. Felici on the 6th March. The doctor suspecting retention of urine, introduced the catheter, and drew off *thirteen or fourteen pints of urine!* This operation, repeated twice in the same day, produced the evacuation of an equal quantity. Next day the symptoms were mitigated:—the catheter was passed twice. The fever and thirst continued—but the pain in the side was gone—the thigh could be moved—and the œdema of the lower extremities greatly diminished. On the 8th a purgative was taken, which procured several watery evacuations—but no urine discharged. The catheter again drew off as large a quantity as on the 6th. About noon labour came on—the fœtus and placenta were thrown off without accident, and there was no subsequent lochia. The catheter was used every two hours on the 9th, 10th, 11th, and 12th. On the 13th the abdominal swelling was entirely gone, as was that of the right thigh. It was not till the 18th that the patient was able to make water naturally, for the first time, and by the 31st was able to lay aside the catheter.

This was one of those cases which we occasionally see of immense distention of the urinary bladder. There was a man last year in St. George's Hospital who was supposed to labour under ascites, and paracentesis abdominis was in contemplation, when the house-surgeon happened to introduce a catheter, and drew off many quarts of water, with immediate reduction of the abdominal swelling.

---

8. *Puerperal Neuritis.** Under this title, some papers have lately been published on the continent, especially by M. Dugés, who endeavours to draw a distinction between neuritis and neuralgia, by symptoms and by dissections. Acute neuritis is so rare and so rarely mortal, that anatomical evidence, on this point, is scanty. Reil, Portal, Breschet, Lobstein, and more especially Martinet, however, have proved the existence of inflammation of nerve, besides several writers in this country, as Sanders, Denmark, Wardrop and others. There is little doubt that both acute and chronic neuritis have often passed under the

* M. Dugés. Revue Medicale, Août, 1824.

names of rheumatism, gout, &c. and, indeed, M. Dugés himself appears to have fallen into this error in more than one instance. We shall cite a case to illustrate this. A woman, about 50 years of age, complained, in the course of the year 1819, of pains in her fingers, accompanied, at first, with a sense of formication, and, afterwards, with a sense of coldness. These pains were constant. The painful parts became tumefied, and with difficulty moved. The pain, stiffness, numbness, and swelling, extended to the hands, and then it was confidently pronounced rheumatic gout. By degrees the malady gained the wrists, the forearms—the elbows, and, in the course of six or eight months, this unhappy woman was reduced to complete helplessness, as well as constant sufferings. The name of the disease was now changed from rheumatism to paralysis, and a host of stimulants were employed without benefit. The complaint extended to the shoulders—marasmus made progress—and this poor woman died in lingering and excruciating sufferings! There can be little doubt, our author thinks, that, had an examination been permitted in this case, neuritis would have been manifest.

In order to develop his ideas respecting puerperal neuritis with clearness, our author endeavours to distinguish five different varieties of the disease under consideration, viz. 1mo. *Simple* or circumscribed neuritis. 2ndo *Œdematous*, that is, where there is a serous effusion not only in the cellular tissue of the nerve itself, but in the surrounding cellular structure. 3tio. *Phlegmonous*, often producing suppuration in the nerve and surrounding parts. 4to. *Œdemato-Phlegmonous*, participating in the characters of the last two varieties. 5to. *Gangrenous*, ending in mortification of the nerve and surrounding tissues. On each of these varieties our author offers some observations, with illustrative examples.

1. *Circumscribed Neuritis*. This variety, he remarks, has almost always been attributed to compression of the nerve—but this does not always hold good. To it has generally been assigned the designation of *neuralgia*, which last, our author thinks, may be distinguished by the remissions or intermissions of pain; whereas, in *neuritis*, the pain is constant. M. Martinet has proved that inflammation of a nerve is not necessarily accompanied by swelling of the organ. This variety often runs its course or is subdued in five or six days—occasioning, at times, only some darting and temporary pains—at others, more acute and lancinating sufferings than the phlegmonous variety. It cedes more readily to sanguineous evacuations than the last-mentioned. Anodynes are of no use in it—but warm baths are of the greatest advantage. The sciatic nerve is the usual seat of this variety—sometimes confined to the pelvic region—sometimes extending to the thigh, the leg, and even to the foot.

2. *Œdematous Neuritis*. This is less frequent than the former variety, and generally less painful. It quickly produces œdema of variable extent, sometimes occupying the whole of the inferior member—always assuming an active character at the beginning, and a passive one towards the conclusion. In this, the pain subsides in a little time, but the œdema continues obstinate, and then generally gives rise to swellings in the lymphatic glands of the groin, if the disease be in the crural nerve. In one dissection, our author found some purulent matter, as well as serous infiltration, in the nerve.

3. *Phlegmonous Neuritis.* This variety is not confined to the lower extremities; but sometimes affects the arms or fore arms of parturient women. Its most frequent seat, however, is the crural, or the sub-pubian nerves. It is often mistaken for common abscess—the nerve escaping the notice of the pathologist, even on dissection. Our author distinguishes it by the pain following the line of the nerve—by being of an extremely severe and insupportable character—by the swelling being prolonged in the direction of the nerve—by the swelling always *preceding* the redness on the skin, and *following* the pain of the part—by the swelling being harder and more unequal than in common phlegmon. On dissection, our author has generally found the nerve infiltrated with concrete pus disseminated among its filaments. Besides this, the surrounding cellular tissue, and sometimes the neighbouring muscles themselves, were infiltrated with purulent matter. At other times, a vast deposit of matter was found disfiguring and confounding all the parts involved in the disease—a state which he thinks most commonly obtains when the disease proves fatal.

4. *Œdemato-Phlegmonous Neuritis.* This is the phlegmatia dolens, about the nature of which so much has been written in this and in other countries. Our author observes, that general opinion attributes phlegmatia dolens to inflammation of the lymphatic system of the parts—and he believes that this opinion may be right, in some cases. But, from what he has seen of the disease, he is fully convinced that, in the majority of cases, the seat of the inflammation is primarily in the nerves, whatever other structures may be involved in the course of the complaint. In this doctrine he appears to be supported by the authority of *Gardien*, who uses the following words:—" si au tiraillement des nerves pendant le travail—si à ce tiraillement qui les rend plus susceptibles de produire, dans les organes auxquels ils se distribuent, un état inflammatoire, ou sur-ajoute la predisposition plus grande que doit produire l'état des couches, on aura une explication naturelle de la frequence de cette affection." This precisely accords with our author's sentiments. He does not notice, and probably is unacquainted with, the doctrine which our able countryman, Dr. Davis, has lately brought forward, respecting the primary seat of the affection. Mauriceau and Casper are quoted by our author as favouring the doctrine of nervous inflammation being the cause of phlegmatia dolens. But it must be confessed that few of the theories of this disease are grounded on dissection—and those who have taken *post-mortem* examinations for their guide, appear to have been still more led astray than those who have framed their opinions from the train of symptoms during life. The way in which pathology has led physicians into error, on this point, as on many others, we have already shewn in several places in this Journal. Our author candidly acknowledges that, in a dissection of phlegdolens, related by Casper, there was neither swelling nor redness of the nerves. There was infiltration of the cellular substance in the neighbourhood of the nerves, and the lymphatics were diseased. No mention is made by our author of the state of the veins in Casper's case, and therefore we may conclude that there was no serious disease in them.

5. *Gangrenous Neuritis.* No example of insulated gangrene of a nerve can be found by our author on record. But then he is inclined to think, with Tommasini, that *inflammation* of nerve is often the cause of *gangrene* in the parts to which the inflamed nerve is distributed. This is Tommasini's doctrine. "Tommasini attribue à l'inflammation des nerves *toutes les phlegmasies gangréneuses.*" This, we think, is carrying theory too far.

Our limits will not permit us to analyse any of the cases brought forward by M. Dugés, in illustration of the foregoing observations. The cases indeed are not very satisfactory ones, and therefore we shall pass them over.

---

9. *Separation of the Ossa Pubis.** It was thought by many, and by Chaussier among the rest, that, in every labour there was a natural separation of the ossa pubis, to a greater or less extent. This opinion, we cannot think, with Dr. Nicholson, is, "at the present time much insisted on." We do not know any living anatomist or accoucheur who entertains such an idea. If indeed such a natural separation was credited, (excepting as an effect of disease, or as a se [illegible]ous accident) why should the proposal and actual execution of an *artificial* separation be brought forward in cases of difficult parturition?—It is sufficient to say that Boër, the Master of the Vienna Hospital, has not seen such a thing in twenty years—nor has it been observed in the Lying-in-Hospital of Dublin, where upwards of three thousand women are annually delivered. We shall therefore come at once to the subject of the present paper.

*Case.* Our author was sent for, on the 3d of December, 1820, to attend a lady, aged 21, in labour of her first child. She was healthy, and of a lively disposition, though delicate. He did not arrive until three hours after delivery; but an intelligent midwife assured him that the labour had not been severe, and that the placenta came away easily. He returned home. Next day she complained of some uneasiness and pain, without particularly describing its locality. The milk was duly secreted—there was no fever—and no apprehension was entertained. On the third day the lady put on her stockings, and moved, with assis-

---

* Dr. A. S. Nicholson. Irish Transactions, Vol. IV.

tance, to the fire-place. On the fourth morning she was totally unable to move her limbs—and informed our author that, while sitting at the fire, the day before, she felt sick, and fell off the low seat on which she was sitting. The nurse found her on the floor in a fainting condition. "In the course of the fifth day she was seized with frequent rigors, so violent as to shake the entire bed; and she complained of excruciating pain at the edge of the os pubis, and along the course of the left thigh. The fainting and rigors returned to such an excess, that I found it necessary to remain with her. Wine, and other stimulants, were given, which soon alleviated these distressing symptoms. Her stomach, however, was at times much disturbed; and she was tormented with noise in her ears, and constant sneezing, which greatly aggravated the pain at the pelvis." The black drop was now of much use in relieving these symptoms. "On putting my finger" says Dr. N. "on the edge of the symphysis pubis, I found it to move distinctly, and a crackling noise was very perceptible to those who were present. She had great pain at the sacrum, and on examining it particularly, I found a tumour on each side of it, about the size of a hazel-nut, hard and circumscribed." Whenever she was moved the pain was agonizing. Our author hinted his suspicions to the family that the pubes were separated, and requested a consultation. Meantime, strict rest was enjoined, and a broad firm bandage was applied round the pelvis, from which great relief was experienced. A solution of muriate of ammonia was applied to the tumours on the sacrum, and they soon disappeared.

"For nearly six weeks she remained perfectly well in her health, and easy in her bed, except when she attempted to move, or to turn on either side, on which occasion she always suffered the most violent pain. She could stretch her feet downwards, but could not draw them up again. She found relief from leaning forwards, and placing her elbows on her knees; and when that position became irksome, she returned to her usual one on her back, where she always felt easy. About this time she menstruated, and though much benefit was expected from this circumstance, yet no alteration took place in her complaint." 86.

Ten weeks after her confinement, Dr. Beatty saw the patient, with Dr. N. and on examination, found all the internal parts healthy and in their natural situation. On considering the seat of pain, however, and the inability of moving her limbs, no doubt was entertained that the symphysis had been separated. The broad bandage was continued, with cold applications to the seat of the pain. A bandage to keep the knees together was also suggested by Dr. Beatty. After five months continuance in this melancholy state, our author was informed by Dr. M'Donnel of Belfast, that he knew a medical gentleman, whose wife had sustained an injury of this kind in a still severer degree; and Dr. M. kindly communicated some of the particulars to our author. It appeared that, for several days before labour came on, the patient suffered much from pain and weakness in her back, and a total inability of moving herself, which caused her labour to be unusually severe. "A crackling noise could be distinctly heard at several yards distance, and not only were the pelvis (ossa ilia) and sacrum separated, but the disease seemed to have extended to the *functions* of all the bones of the pelvis." Many medical men were consulted, and many remedies tried, of course, but at the end of seven months no improvement had taken place, and her situation was deplorable. She now tried the shower-bath, and the good effects were soon apparent; for, in a few weeks, she was able to walk on crutches, and in two months to go up stairs. In three months she was fully restored to the use of her limbs, and has since had a living child.

The results of the above case were communicated to Dr. Nicholson's patient, and she gladly commenced the shower-bath. Before the expiration of a month, great relief from pain was experienced, and the patient was once more able to pull on her stockings and draw up her feet. She continued free from pain and in good health till December (12 months from her confinement) when she was injudiciously removed in a chaise from her father's to her husband's house. All the unpleasant symptoms now returned, accompanied with great pain, and the crackling of the pubis. She continued in a miserable state, without hopes of recovery; but on resuming the shower-bath and belt, she evidently improved. She was enabled to turn herself in bed—and at length was able to be wheeled about the garden in a little carriage. By the last accounts which our author had, she was able to shuffle across the room by the aid of crutches.

